# Ketogenic interventions in mild cognitive impairment, Alzheimer's disease, and Parkinson's disease: A systematic review and critical appraisal

**DOI:** 10.3389/fneur.2023.1123290

**Published:** 2023-02-09

**Authors:** Jeffrey L. B. Bohnen, Roger L. Albin, Nicolaas I. Bohnen

**Affiliations:** ^1^Department of Neurology, University of Michigan, Ann Arbor, MI, United States; ^2^Neurology Service and GRECC, VA Ann Arbor Healthcare System, Ann Arbor, MI, United States; ^3^Morris K. Udall Center of Excellence for Parkinson's Disease Research, University of Michigan, Ann Arbor, MI, United States; ^4^Parkinson's Foundation Research Center of Excellence, University of Michigan, Ann Arbor, MI, United States; ^5^Department of Radiology, University of Michigan, Ann Arbor, MI, United States

**Keywords:** Alzheimer's disease, mild cognitive impairment, Parkinson's disease, ketogenic, effectiveness

## Abstract

**Background:**

There is increasing interest in therapeutic ketosis as a potential therapy for neurodegenerative disorders–in particular, mild cognitive impairment (MCI), Alzheimer's disease (AD), and Parkinson's disease (PD)–following a proof-of-concept study in Parkinson's disease published in 2005.

**Methods:**

To provide an objective assessment of emerging clinical evidence and targeted recommendations for future research, we reviewed clinical trials involving ketogenic interventions in mild cognitive impairment, Alzheimer's disease, and Parkinson's disease reported since 2005. Levels of clinical evidence were systematically reviewed using the American Academy of Neurology criteria for rating therapeutic trials.

**Results:**

10 AD, 3 MCI, and 5 PD therapeutic ketogenic trials were identified. Respective grades of clinical evidence were objectively assessed using the American Academy of Neurology criteria for rating therapeutic trials. We found class “B” evidence (probably effective) for cognitive improvement in subjects with mild cognitive impairment and subjects with mild-to-moderate Alzheimer's disease negative for the apolipoprotein ε4 allele (APOε4-). We found class “U” evidence (unproven) for cognitive stabilization in individuals with mild-to-moderate Alzheimer's disease positive for the apolipoprotein ε4 allele (APOε4+). We found class “C” evidence (possibly effective) for improvement of non-motor features and class “U” evidence (unproven) for motor features in individuals with Parkinson's disease. The number of trials in Parkinson's disease is very small with best evidence that acute supplementation holds promise for improving exercise endurance.

**Conclusions:**

Limitations of the literature to date include the range of ketogenic interventions currently assessed in the literature (i.e., primarily diet or medium-chain triglyceride interventions), with fewer studies using more potent formulations (e.g., exogenous ketone esters). Collectively, the strongest evidence to date exists for cognitive improvement in individuals with mild cognitive impairment and in individuals with mild-to-moderate Alzheimer's disease negative for the apolipoprotein ε4 allele. Larger-scale, pivotal trials are justified in these populations. Further research is required to optimize the utilization of ketogenic interventions in differing clinical contexts and to better characterize the response to therapeutic ketosis in patients who are positive for the apolipoprotein ε4 allele, as modified interventions may be necessary.

## 1. Introduction

Therapeutic ketosis has been successfully used in neurology in the management of treatment-refractory pediatric epilepsy for about a century (1). Despite this long-standing experience with the ketogenic diet, evidence supporting the use of ketogenic interventions for other neurological conditions has been slow to emerge. It was not until recently that a small open-label study reported preliminary evidence that a ketogenic diet may benefit patients with PD (2). This study was the first of an increasing body of literature assessing the efficacy of ketogenic interventions in neurodegenerative disorders.

A considerable body of data suggests that therapeutic ketosis would be a useful intervention for PD, AD, and MCI. Mitochondrial dysfunction is widely thought to be a major pathogenic mechanism in PD. Neuronal metabolism of ketone bodies may bypass the mitochondrial complex I deficiency implicated as a major feature of PD mitochondrial dysfunction and mitigate neuronal dysfunction in PD (3–6). In AD, studies comparing regional brain metabolism, as measured by [^18^F]fluorodeoxyglucose (FDG) positron emission tomography (PET), with regional synaptic terminal density measured with [^11^C]UCB-J PET suggest relative preservation of synapses in neocortical hypometabolic regions (7). Similar findings are described in a PD animal model (8). In AD, analogous results were obtained with comparative FDG PET and assessment of regional brain ketone body metabolism measured with [^11^C]AcAc PET (9). The cumulative results suggest that both AD and PD are characterized by metabolically impaired synapses whose functions might be improved by provision of alternative energy substrates. Such interventions might be particularly effective as a form of secondary prevention in prodromal phases of neurodegenerative syndromes, such as MCI (10, 11). Progressive deficits in brain energy metabolism have been implicated in the progression from MCI to AD, suggesting a potential role for metabolic interventions such as therapeutic ketosis in modifying disease progression (12, 13). Given the pleiotropic effects of ketogenic interventions, however, the relative importance of differing mechanisms may vary depending on the stage of disease and clinical context.

To provide some context, it is important to recognize two schools of thought. One is the popular ‘alternative fuel' hypothesis in the setting of insulin resistance in the brain as manifested by glucose metabolic reductions in patients with advanced cognitive decline. The second one is the ‘signaling' hypothesis, in which direct and epigenetic effects of ketone bodies may drive anti-inflammatory, anti-oxidant, and anti-neurodegenerative (e.g., anti-amyloid) mechanisms, in addition to various other mechanisms that include modulation of neurotransmission and neuroplasticity (14). The ‘alternative fuel' hypothesis would be most applicable to patients with more severe cognitive deficits as reflected by more severe and more extensive glucose metabolic deficits on FDG PET (9, 15–17). In contrast, the ‘signaling' hypothesis may bear more relevance for earlier stages of neurodegeneration such as MCI, where glucose metabolic FDG PET brain reductions are less severe and less extensive (9, 11, 17–20). In this respect, therapeutic ketosis in earlier disease stages may potentially exert a more disease-modifying or even secondary preventive effect reflected by changes in signaling pathways, biomarkers, and disease progression, as opposed to providing more immediate symptomatic relief. More research, however, is needed to elucidate this.

At the time of this writing, various methods of inducing ketosis have been developed. In addition to traditional fasting and the ketogenic diet, alternative approaches include modified ketogenic diets (which involve various macronutrient ratios limiting carbohydrates and excess protein) as well as exogenous ketone supplements. Ketone supplements may include synthesized ketones bound to a ketone precursor in an ester compound (referred to as “ketone esters”), synthesized ketones bound to a salt (referred to as “ketone salts”), or a combination thereof. It is also possible to induce ketonemia *via* ingestion of ketone precursors such as medium-chain triglycerides (MCTs), which are found naturally in coconut oil and comprised of caproic acid (C6), caprylic acid (C8), capric acid (C10), and lauric acid (C12). Of particular relevance to discussion of clinical trials assessing ketogenic interventions for neurodegenerative disorders is the oral ketogenic compound, AC-1202 (marketed as “Axona”), which includes caprylic acid, a medium-chain triglyceride. Vigorous exercise provides another pathway to ketosis, with potential for synergistic effects when combined with other interventions. Here, we review the literature for a variety of ketogenic interventions in the context of MCI, AD, and PD.

The purpose of this study was to review the current level of evidence supporting the clinical utility of ketogenic interventions in MCI, AD, and PD. We also reviewed biomarker outcomes and effects of normal aging to provide a mechanistic perspective on the role of therapeutic ketosis in neurodegeneration. Moreover, we stratified outcomes in AD based on apolipoprotein ε genotype, which may play a modulating role in ketone metabolism (21–23). Building on the literature gaps and remaining mysteries identified in this review, we also include hypotheses and directions for future research.

## 2. Methods

References included in this review were identified *via* advanced search in PubMed.gov, with “ketogenic,” “neurodegenerative,” and “disorders” included among key terms of interest. Additional references were extracted from the reference lists of selected articles. We restricted our search to articles published in English. Our initial search generated 237 preliminary results, after which the final reference list was generated on the basis of relevance to this review and by using the PubMed.gov “Clinical Trial” filter to exclude non-interventional studies. The final review was thus narrowed down to 25 studies, with priority given to randomized, controlled clinical trials investigating the effects of ketogenic interventions for patients with MCI, AD, and PD. In light of the central role that aging plays in neurodegenerative processes, we also reviewed some studies assessing ketogenic interventions in the setting of normal aging. The size, heterogeneity of interventions, and heterogeneity of outcome measures of the identified studies precluded meta-analysis.

The American Academy of Neurology (AAN) Classification of Evidence framework was then applied to our literature review. To provide a brief overview of this framework, individual studies can be classified as Class I, Class II, Class III, or Class IV (in descending order of evidence strength) using a set of criteria that takes into account a publication's methodology, outcomes, and transparency. In turn, recommendations for an intervention as a whole can be synthesized based on the collective quality of available evidence, with possible grades for a recommendation spanning: “A” (established as effective), “B” (probably effective), “C” (possibly effective), and “U” (data inadequate or conflicting). The AAN Classification of Evidence framework is included as Supplementary material.

## 3. Results

Table 1 provides an overview of identified clinical trials in AD, MCI, and PD with information on study population, type of ketogenic intervention, duration of treatment, randomization, blinding, primary and secondary outcome parameters, apolipoprotein ε genotype (if assessed), and relevant biomarker trends, including changes in blood β-hydroxybutyrate (β-HB) concentration as well as brain ketone body uptake if magnetic resonance spectroscopy (MRS) or PET imaging was utilized. Each study was assigned a level of evidence for therapeutic efficacy based on AAN Classification of Evidence criteria, after which the AAN Classification of Recommendations criteria were applied to provide collective grades for clinical indications based on available evidence to date (summarized in Table 2). Using this classification system, ketogenic interventions for MCI, AD, and PD can be graded as follows, with the rationale for each grade detailed in the Discussion.

**Table 1:** Literature review of ketogenic interventions for MCI, AD, PD, and aging.

**Study**	**Condition**	**Methods**	**Phase**	**Primary outcome measure(s)**	**Positive outcomes**	**Negative outcomes**	**Apoε4**	**Protein biomarkers**	**Ketone blood levels**	**Lipid levels**	**Glucose levels**	**AAN level of** **evidence**
Krikorian et al. (34)	23 older adults with MCI	Randomized to KD vs. high-carb diet for 6 wk	2	Cognition	Improved verbal memory (*p =* 0.01)	N/A	N/A	N/A	Positively correlated with memory improvement	N/A	Fasting glucose levels ↓	Class II
Fortier et al.^*^ (35)	52 subjects with MCI	Blindly randomized to 30 g kMCT daily vs. placebo for 6 mo	2	Cognition, brain AcAc and FDG PET, MRI	Brain ketone metabolism increased by 230% for kMCT group (*p < * 0.001). Improvements in cognitive tests positively a/w ↑ brain ketone metabolism	Adverse effects primarily GI upset	No significant effect of APOε4 status on ketosis or cognitive outcomes, though not adequately powered to assess this	N/A	Mean [ß-HB] significantly increased to 0.543 mmol/L (*p =* 0.001)	N/A	Brain glucose uptake unchanged	Class II
Neth et al. (19)	20 subjects with SMC or MCI	Randomized crossover: MMKD vs. LF-AHAD for 6 wk (with 6 wk washout period prior to crossover)	2	Cognition, brain AcAc and FDG PET, MRI	Improved metabolic indices. Increased cerebral perfusion among subjects with MCI (no change in perfusion among subjects with SMC). Increased cerebral ketone body uptake (assessed *via* PET tracer).	Memory improved w/ both diets (though practice effects may have played a role), with relatively greater effect size for MMKD. Not all cognitive outcome parameters reached significance	Higher prevalence of APOε4 among MCI Pts (44% vs 20% for SMC); lower ketone levels in MCI group despite similar dietary compliance; MCI group also had higher fasting insulin and TG levels at baseline	↑ CSF Aβ42 ↓ CSF Tau ↓ CSF Neurogranin	Significantly increased (mean [ß-HB] of 0.7 mmol/L) w/ ↑ cerebral uptake of ketones	↑ LDL ↓ VLDL ↓ TG (*p < * 0.05)	Significant reduction in HbA1c	Class II
Fortier et al.^*^ (36)	122 subjects with MCI	Blindly randomized to 30 g kMCT daily vs. placebo for 6 mo	2	Cognition, brain AcAc and FDG PET, MRI	Significant Improvements in cognitive tests compared to placebo. Some cognitive outcomes also positively correlated with ↑ brain ketone metabolism	Adverse effects primarily GI upset	No significant effect of APOε4 status on ketosis or cognitive outcomes, though not adequately powered to assess this	Measured plasma amyloid beta (Aβ), but did not see any differences between groups or after the kMCT or placebo	Mean [ß-HB] increased significantly to >0.4 mmol/L Immediately after administration of kMCT (*p < * 0.0001)	Total cholesterol increased in treatment group, but stayed within clinical reference range	Blood glucose increased in treatment group, but stayed within clinical reference range	Class II
Roy et al.^*^ (37)	37 subjects with MCI	Blindly randomized to 30 g kMCT daily vs. placebo for 6 mo	2	Processing speed, brain AcAc PET	Ketone uptake was increased in kMCT group 2.5-3.2-fold in white matter areas of interest. Improvements in processing speed, which were positively associated with white matter uptake	N/A	N/A	N/A	Mean [ß-HB] increased significantly to 0.572 mmol/L 2 h after administration of kMCT (*p < * 0.0001)	N/A	kMCT → nearly 3-fold increase in white matter ketone uptake, but there was no significant effect on glucose uptake	Class II
Myette-Côté et al.^*^ (38)	39 subjects with MCI	Blindly randomized to 30 g kMCT daily vs. placebo for 6 mo	1	Cardio-metabolic and inflammatory biomarkers	MCT supplementation was assessed for its effect on cardiometabolic and inflammatory markers, revealing a reassuring safety profile	Increased IL8 observed in MCT group (unclear clinical significance) w/ no significant effects on other inflammatory markers	N/A	Measured plasma amyloid beta (Aβ), but did not see any differences between groups or after the kMCT or placebo	kMCT group showed significant increase in total ketones post-intervention (+0.416 mmol/L)	Cholesterol and TG levels all remained within normal range throughout intervention	No significant change in HbA1c	Class II
Roy et al.^*^ (39)	32 subjects with MCI	Blindly randomized to 30 g kMCT daily vs. placebo for 6 mo	2	Brain MRI connectivity, brain AcAc and FDG PET	kMCT supplementation a/w increased functional connectivity in the dorsal attention network (DAN), which correlated w/ improvement in cognitive tests. Ketone uptake in DAN cortical areas significantly increased in kMCT group, which was directly a/w improvements in DAN functional connectivity	N/A	N/A	N/A	N/A	N/A	After the intervention, mean DAN white matter glucose uptake increased by 8% in the kMCT group (*p =* 0.039), with no change in the placebo group	Class II
Henderson et al. (24)	152 subjects with mild-to-moderate AD	Randomized, double-blinded, placebo-controlled trial of an oral ketogenic compound (AC-1202); taken daily for 90 d	2	Cognition and safety	Improvement in cognitive scores compared to placebo (*p < * 0.05)	GI adverse effects most common; 24.4% of subjects in experimental group reported diarrhea	Cognitive benefits only observed among APOε4- participants, which correlated with ↑ serum ß-HB levels; no correlation b/n cognitive scores and serum ß-HB levels among APOε4+ subjects	N/A	Post-dose ß-HB levels significantly elevated to mean of 0.36-0.39 mmol/L for experimental group as compared to placebo, though pre-dose ß-HB levels did not significantly differ at any time point	N/A	N/A	Class II
Taylor et al. (25)	15 subjects with AD	Single arm: KD + MCT for 3 mo, followed by 1 mo washout (normal diet resumed)	1	Cognition	Improved cognitive scores during diet among completers (*p =* 0.02); scores reverted after washout	High dropout rate among those w/ moderate AD, citing caregiver burden GI upset, primarily MCT-related	N/A	N/A	Serum ß-HB levels significantly increased during diet (peaked in first month at average of 0.52 mmol/L)	↑ HDL, LDL, and total cholesterol (though did not reach statistical significance)	Remained stable	Class III
Croteau et al. (26)	20 subjects with mild-to-moderate AD	Consumed two different kMCT supplements: C8C10 30 g daily for 1 mo followed by 1 mo washout then C8 30 g daily for 1 mo	2	Brain AcAc and FDG PET	Brain ketone metabolism doubled for both supplements. Slope of relationship b/n plasma ketones and brain ketone uptake same as in healthy young adults	N/A	N/A	N/A	Mean [ß-HB] significantly increased to 0.57 mmol/L post-C8; mean [ß-HB] significantly increased to 0.46 mmol/L post-C8C10	↑ TG No significant difference in HDL, LDL, and total cholesterol	Total brain metabolism ↑ increased 2/2 ketone metabolism w/ brain glucose metabolism unchanged No significant change in HbA1c	Class III
Torosyan et al. (27)	16 subjects with mild-to-moderate AD	Small double-blinded, placebo-controlled RCT. 14 subjects assigned to caprylidene (ketone precursor); 2 assigned to placebo. Intervention taken for 45 d	2	Cerebral blood flow (*via* O-water PET)	Daily ingestion of caprylidene a/w increased regional cerebral blood flow	N/A	Increased regional cerebral blood flow observed only among APOε4- subjects. No significant effect for APOε4+ subjects	N/A	N/A	N/A	N/A	Class III
de la Rubia Ortí et al. (28)	44 subjects with AD	Randomized to Mediterranean diet enriched with coconut oil vs. isocaloric control diet for 21 d	2	Cognition	Improved cognitive function (more pronounced in women w/ less severe disease)	N/A	N/A	N/A	N/A	N/A	N/A	Class II
Ota et al. (29)	20 subjects with mild-to-moderate AD	SS1: Double-blinded RCT comparing MCT formula to placebo w/ cognitive testing and ketone blood level measurement 2 h after single administration SS2: 3-mo open-label study of daily MCT formula ingestion, measuring cognitive function over time	2	Cognition	Significant improvements in cognitive scores for open-label trial of daily supplementation w/ MCT formula (*p < * 0.01), though potentially confounded by training effects in addition to lack of blinding, with subjects serving as own controls	No significant difference in cognitive scores following single administration of supplement. No significant longitudinal increase in endogenous baseline ketone blood level (measured in setting of withholding daily MCT formula at 4, 8, and 12 wks)	Did not assess. Authors acknowledged this may have been a confounding factor	N/A	MCT formula significantly increased [ß-HB] to mean of 0.4709 mmol/L when measured 2 h after administration (*p < * 0.001)	Only measured at baseline	Only measured at baseline	Class III
Xu et al. (30)	53 subjects with mild-to-moderate AD	Double-blinded, randomized, placebo-controlled crossover study comparing 17.3 g/d of MCTs to canola oil for 30 d	2	Cognition	Significant improvements in cognitive scores compared to placebo	Changes in ADL scores did not significantly differ between MCT and placebo groups	Statistically significant cognitive benefits observed only among APOε4- Pts, though subtle signal suggesting intervention may stabilize cognitive decline among APOε4+ Pts	N/A	After 30 d of MCT intervention, [ß-HB] was 129% higher at baseline in subjects fasting for more than 12 hr (measured prior to MCT dosing): attained mean fasting [ß-HB] of 0.09015 mmol/L (*p < * 0.01 compared to placebo)	↑ total cholesterol ↑ HDL (*p < * 0.01)	N/A	Class I
Henderson et al. (31)	413 subjects with mild-to-moderate probable AD	Randomized, double-blinded, placebo-controlled trial of an oral ketogenic compound (AC-1204); taken daily for 26 wk	3	Cognition and safety	N/A	Failed to improve cognitive or functional capacity. GI upset most common adverse effect	Primary analysis focused on APOε4- Pts, but did not demonstrate significant benefit	N/A	Mean post-dose [ß-HB] ranging from 0.109-0.272 mmol/L	N/A	N/A	Class II design; however, did not reach endpoint
Phillips et al. (32)	26 subjects with AD	Randomized crossover: KD vs. control diet for 3 mo (with 1 mo washout prior to crossover)	2	Quality of life, ADL capacity, and cognition	Improved quality of life (*p =* 0.02) and ADL capacity (*p < * 0.01)	Cognitive scores also increased, but not significant (*p =* 0.24)	Authors concluded that carrier status may be a/w ↓ cognitive benefit of KD, though acknowledged potential confounding factors	N/A	Mean serum ß-HB level of 0.95 mmol/L during diet (significantly increased)	↑ HDL, LDL, and total cholesterol (*p < * 0.05)	Significant reduction in HbA1c	Class II
Juby et al. (33)	20 subjects with mild-to-moderate AD	8-month randomized, double-blinded, placebo-controlled, crossover study comparing 42 g/d of MCT oil to olive oil (phase 1), followed by open-label extension of MCT oil by all subjects for 7 months (phase 2)	2	Cognition and safety	Overall, at the conclusion of the extended 15-month protocol (crossover phase followed by open-label extension of MCT for all participants), 80% of subjects demonstrated either cognitive improvement or slowed rates of cognitive decline (thus considered responders to stabilization effects)	During RCT phase, cognitive outcomes for experimental arm did not reach statistical significance. Adverse events were primarily GI-related	No measurable effect of APOε4 status on response to MCT oil	N/A	Average fasting (pre-intervention) baseline ß-HB level was 0.19 mmol/L, and at study completion was 0.22 mmol/L (no significant change)	No significant change in total cholesterol, LDL, and TG. Weak association noted for lower LDL and total cholesterol in those consuming higher doses of MCT oil	No impact on HbA1c and fasting insulin	Class III
VanIttalie et al. (2)	7 subjects with PD	Open-label feasibility study of strict KD for 28 days	1	UPDRS scores and lipid levels	5/7 subjects completed the study, though 2/5 completers had occasional lapses in adherence to KD → all 5 completers showed improved total UPDRS scores and improved motor subscores	One subject was unable to prepare diet; another withdrew for unrelated reasons. Potential confounding factors (lack of control group) make it difficult to interpret results beyond confirmation of feasibility for future research	N/A	N/A	Notably high levels of serum ß-HB (mean of 6.6 mmol/L among three most adherent subjects)	Serum cholesterol levels were not significantly different for 4/5 subjects; for one subject who had high cholesterol at baseline, total cholesterol increased 30% after 28 days	N/A	Class III
Phillips et al. (41)	47 subjects with PD	Randomized to less stringent KD (as compared to the diet used by VanIttalie et al.) vs. LFD for 8 wk	2	UPDRS scores	41% improvement in UPDRS I scores (non-motor) (*p < * 0.01)	Intermittent exacerbation of PD tremor and/or rigidity in KD group	N/A	N/A	Mean serum [ß-HB] significantly increased to 1.15 mmol/L	KD significantly increased HDL, LDL, and total cholesterol (*p < * 0.001)	Significant reduction in mean weekly bedtime glucose levels (*p =* 0.001)	Class II
Krikorian et al. (42)	14 subjects with MCI in the setting of PD	Randomized to KD vs. control diet for 8wk	2	Cognition, motor symptoms (*via* UPDRS-III)	Improvement in memory	No improvement in motor function	N/A	N/A	Mean serum [ß-HB] significantly increased to 0.31 mmol/L	N/A	Fasting insulin significantly declined, whereas fasting glucose did not	Class III
Koyuncu et al. (43)	74 subjects with PD who reported a voice disorder related to their disease	Randomly assigned to KD vs. regular diet for 3 months	2	Vocal quality	KD significantly improved voice quality	GI upset in 5 subjects for KD group, compared to 2 in control group	N/A	N/A	N/A	N/A	N/A	Class II
Norwitz et al. (40)	14 subjects with Hoehn and Yahr stage 1-2 PD	Randomized, placebo-controlled, crossover study comparing acute effect of ketone ester drink vs. isocaloric, taste-matched placebo drink on exercise endurance	1	Exercise endurance	Participants sustained exercise for 24% longer after consuming the ketone ester drink compared to the isocaloric control drink	N/A	N/A	N/A	Mean serum [ß-HB] increased significantly to 3.5 mmol/L within 30 min of ketone ester consumption	N/A	Less significant rise in glucose associated with formula that contained ketone ester	Class I (Exemplary trial design, though this study did not assess direct effects on clinical symptoms)
Freemantle et al. (60)	32 healthy subjects stratified by age group	A ketogenic meal was consumed with various metabolic markers tracked to assess potential interaction effects between age and ketone metabolism	1	Blood ß-HB, glucose, and lipid levels	Elderly people in relatively good health have similar capacity to produce ketones and oxidize ß-HB as compared to middle-aged or young adults	N/A	APOε4 carriers had significantly elevated cholesterol levels, but no significant differences in other metabolites	N/A	Mean serum ß-HB level increased similarly for all three groups	No significant acute change in lipid levels	Among elderly subjects, glucose was oxidized more rapidly as compared to healthy middle-aged adults	Class III
Abe et al. (61)	64 elderly nursing home residents with frailty	Randomly assigned to receive 6 g/d MCTs, 6 g/d MCTs plus Vitamin D3 and L-leucine, or 6 g/d LCTs for 3 mo	2	Cognition	Significant improvement in MMSE for subjects receiving MCTs compared to LCTs (*p < * 0.001), though only partially blinded (examiners aware of subject group)	N/A	N/A	N/A	N/A	N/A	N/A	Class III
Mujica-Parodi et al. (62)	42 adults were assessed *via* fMRI for network stability (marker of brain aging)	Two experiments conducted involving: (1) comparison of standard diet to overnight fasting and ketogenic diet conditions (KD was sustained for 1 wk prior to fMRI) (2) exogenous ketone ester drink was also compared to calorie-matched glucose drink, with network stability measured as primary outcome	2	fMRI network stability	Networks were destabilized by glucose and stabilized by ketones, regardless of whether ketosis was achieved *via* ketogenic diet or exogenous ketone ester. MRS showed ketones reaching peak concentrations in brain at ~30 min post-dose. Network stability validated as a marker of aging; destabilization effects emerged at 47 years, with most rapid degeneration occurring at 60 years	N/A	N/A	N/A	Ketone ester raised [ß-HB] to mean of 3.5 mmol/L (measured 50 min after administration)	N/A	Ketone ester consumption associated with acute reduction in blood glucose (mean reduction of 18 mg/dL, as measured 50 min after consumption of ketone ester)	Class II

* Several publications were derived from the multi-phase BENEFIC trial dataset.

a/w, associated with; AAN, American Academy of Neurology; AD, Alzheimer's disease; ADL, Activities of Daily Living; APOε4, Apolipoprotein ε4; ß-HB, beta-hydroxybutyrate; CSF, cerebrospinal fluid; FA, first author; fMRI, functional Magnetic Resonance Imaging; GI, gastrointestinal; HDL, high-density lipoprotein; IL, interleukin; KD, ketogenic diet; kMCT, ketogenic medium-chain triglycerides; LCT, long-chain triglyceride; LDL, low-density lipoprotein; LF-AHA, low-fat American Heart Association diet; LFD, low-fat diet; MCI, mild cognitive impairment; MCT, medium-chain triglyceride; MMKD, modified Mediterranean ketogenic diet; MMSE, Mini-Mental Status Exam; mo, month; MRS, Magnetic Resonance Spectroscopy; N/A, not applicable; PD, Parkinson's disease; PMID, PubMed reference number; SMC, subjective memory complaints; SS, Sub-study; TG, triglycerides; UPDSR, Unified Parkinson's Disease Rating Scale; VLDL, very-low-density lipoprotein; 2/2, secondary to.

**Table 2:** Collective strength of evidence to date (per AAN criteria): ketogenic interventions in AD, MCI, and PD.

For cognitive improvement among patients with mild-to-moderate AD who are APOε4-	“B” (probably effective)
For cognitive improvement among patients with mild-to-moderate AD who are APOε4+	“U” (unproven)
For cognitive improvement among patients with MCI^*^	“B” (probably effective)
For improvement of non-motor features in PD	“C” (possibly effective)
For improvement of motor features in PD	“U” (unproven)

* While there are consistent Class II studies demonstrating cognitive benefits of ketogenic interventions among subjects with MCI, some of these studies were not sufficiently powered to assess the potential modulating effect of APOε4 status on ketosis or therapeutic outcomes.

Our review identified 10 studies in AD, with levels of evidence ranging from Class I to Class III and an overall AAN-based grade of “B” (probably effective) (24–33). In 2009, Henderson et al. (24) found that an oral ketogenic compound, AC-1202 (a medium-chain triglyceride composed of glycerin and caprylic acid [C8]), was superior to placebo in improving cognitive outcomes among subjects with mild-to-moderate AD negative for the apolipoprotein ε4 allele (APOε4-) (24). A subsequent clinical trial by the same group of investigators using an altered formulation, AC-1204 (a proprietary formulation designed to improve tolerability and comprised of 50% caprylic triglyceride by weight), failed to achieve its primary endpoint in a phase 3 clinical trial (31). The use of the new formulation resulted in substantially lower blood levels of β-HB, which may explain the unsuccessful replication of earlier study findings. Similarly, Xu et al. (30) reported significant improvements in cognition associated with supplementation of medium-chain triglyceride (MCT) oil as compared to placebo among individuals with mild-to-moderate AD who were APOε4- (30). This was the only AD study meeting Class I evidence criteria for therapeutic efficacy. Other studies provided Class II evidence for the efficacy of other ketogenic interventions in AD, such as a ketogenic diet or a Mediterranean diet enriched with coconut oil (28, 32). Two studies utilized PET imaging to assess trends in cerebral metabolism. Croteau et al. (26) found that supplementation with an MCT formula (comprised of 55% caprylic acid [C8] and 35% capric acid [C10]) increased total brain metabolism in AD (a trend driven by increased ketone body uptake, as glucose uptake remained unchanged) (26). Torosyan et al. (27) found that daily ingestion of caprylidene (a medium chain triglyceride of caprylic acid [C8]) was associated with increased regional cerebral blood flow among subjects with mild-to-moderate AD who were APOε4-, but no significant blood flow effects were seen for subjects who were APOε4+ (27).

Our review identified three clinical trials in MCI (with several publications derived from the multi-phase BENEFIC trial dataset) (19, 34–39). The BENEFIC trial provided Class II evidence for the efficacy of a ketogenic MCT formula (a 12% emulsion of Captex 355, comprised of 60% caprylic acid [C8] and 40% capric acid [C10], in lactose-free skim milk) in MCI (35, 36). Several biomarker studies were subsequently derived from this dataset, revealing the following trends with MCT supplementation: increased brain ketone uptake, which was associated with improvements in processing speed (37); a reassuring cardiometabolic and inflammatory safety profile (38); and increased functional connectivity in the dorsal attention network, which was associated with improved performance on cognitive tests (39). Two smaller Class II trials provided further evidence for the efficacy of a ketogenic diet or a modified Mediterranean ketogenic diet in MCI (19, 34). Collectively, in light of multiple consistent Class II studies, the existing literature supports an overall AAN-based grade of “B” (probably effective) for ketogenic interventions in MCI.

Our review identified five studies in PD, with only one Class I study conducted by Norwitz et al. (40) (a study focused on exercise endurance and not directly assessing characteristic clinical features) (40). The other studies identified for PD primarily represented Class III evidence, with one Class II clinical trial supporting the efficacy of a ketogenic diet for non-motor features in PD (2, 41–43). The literature to date supports an overall AAN-based grade of “C” (possibly effective) for non-motor features in PD, with more research needed to assess the utility of ketogenic interventions in treating motor features.

## 4. Discussion

### 4.1. Alzheimer's disease

To date, the strongest evidence for therapeutic ketosis in neurodegenerative disorders supports a role for cognitive improvement in AD and MCI. Henderson et al. (24) conducted a controversial clinical trial in 2009, finding that an oral ketogenic compound, AC-1202 (a caprylic acid [C8] triglyceride), was superior to placebo in improving cognitive outcomes among subjects with mild-to-moderate AD who were APOε4- (24). These phase 2 trial results appeared promising and were used to justify the marketing of AC-1202 (commercially known as “Axona”) as a medical food. This designation led the FDA to issue a warning letter in 2013, asserting that Axona was incorrectly labeled as a medical food and should be held to a more stringent burden of evidence as an investigational new drug. An updated formulation, AC-1204, subsequently failed to achieve its primary endpoint in a phase 3 clinical trial, which could have been related to sub-optimal levels of blood ketone bodies secondary to limited bioavailability (31). Whereas, the AC-1202 formulation was associated with significant elevations in blood ketone bodies (with a mean post-dose serum β-HB concentration of 0.36–0.39 mmol/L), the AC-1204 formulation achieved a lower mean post-dose serum β-HB concentration of 0.109–0.272 mmol/L.

Despite the media backlash against the controversial marketing of Axona, these clinical trials represented advances in this field of research. To date, the Henderson et al. (24) trial is among the most highly powered studies of ketogenic interventions in neurodegenerative disorders with 152 enrolled subjects. The trial employed a randomized, double-blinded design, comparing a ketogenic intervention to placebo *via* objective outcome assessment and intention-to-treat analysis. Under strict AAN criteria, however, the trial experienced dropout rates that preclude classification as Class I evidence (at least 80% of enrolled subjects must complete a study for consideration as Class I evidence). A total of 52 subjects were lost to follow-up or discontinued the protocol (a completion rate of 65.8%), resulting in a Class II classification.

An important takeaway from the Henderson et al. (24) dataset was the conclusion that the benefits derived from ketogenic intervention may be significantly modulated by APOε4 genotype. Henderson et al. (24) observed no significant cognitive benefit associated with AC-1202 supplementation among participants who were APOε4+, while participants who were APOε4- demonstrated significant cognitive improvements. This general trend was supported by limited evidence in subsequent studies. A small, randomized crossover study comparing a ketogenic diet to a control diet in 26 individuals with AD suggested that ketone metabolism may be less beneficial for APOε4 carriers, though the authors acknowledged potential confounding factors affecting their dataset (32). In 2018, Torosyan et al. (27) performed a small, double-blinded, randomized controlled trial comparing caprylidene (a caprylic acid [C8] triglyceride) to placebo in 16 subjects with mild-to-moderate AD, with regional cerebral blood flow assessed as the primary outcome. Increased left superior lateral temporal cortical, left inferior temporal cortical, anterior cerebellar, and hypothalamic blood flow were observed only among subjects who were APOε4-, with no significant effect observed among subjects who were APOε4+ (27).

In 2020, Xu et al. (30) conducted a double-blinded, randomized controlled trial comparing 17.3 g/d of medium-chain triglyceride (MCT) oil to placebo in 53 subjects with mild-to-moderate AD (30). MCT oil supplementation was associated with statistically significant cognitive improvements compared to placebo, but these results were observed only among subjects who were APOε4-. A close look at this dataset reveals a subtle signal suggesting the possibility that MCT oil may preserve cognitive function among APOε4+ patients, as APOε4+ participants receiving MCT oil demonstrated marginal cognitive improvement (i.e., stabilization) while APOε4+ participants receiving placebo demonstrated cognitive decline, though this effect was not statistically significant. Only three of the 49 subjects in this dataset were APOε4+, so any analysis of APOε4+ participants in this sample lacks statistical power, precluding the validity of any interpretations beyond hypothetical reasoning. Albeit with methodological limitations, these cumulative findings suggest the possibility that individuals who are APOε4+ may derive relatively less benefit (if any at all) from ketogenic interventions, at least when dosed equivalently to individuals who are APOε4-. APOε4 expression has been associated with inhibition of the PPAR-γ/PGC-1α signaling pathway, deficits in glycolysis and mitochondrial respiration, enhanced neuroinflammation, and dysregulated lipid metabolism (23, 44–47). Although more data is needed to better characterize the potential modulating role of APOε4 status in therapeutic ketosis, some authors have hypothesized that altered metabolism in the setting of ε4 allele expression may limit the body's capacity to metabolize ketones (in turn, modulating the potential neurological benefits derived from ketogenic interventions) (48, 49).

In contrast, a small, recent clinical trial (*n* = 20) found that subjects with mild-to-moderate APOε4+ AD responded to an MCT oil intervention (33). The trial design included two phases, the first of which was an eight-month, randomized, double-blinded, placebo-controlled crossover study, in which each arm received MCT oil vs. olive oil (placebo) for 4 months prior to crossover. During this phase, cognitive outcomes for the experimental arm did not reach statistical significance. The second phase of the trial involved an open-label extension of MCT oil among all participants for seven months, after which the cognitive test battery was repeated. Overall, at the conclusion of this extended protocol (after completing the crossover trial and the seven-month open-label MCT extension) 80% of the subjects demonstrated either cognitive improvement or slowed rates of cognitive decline, as calculated *via* Mini-Mental State Exam (MMSE) score trends. 19 participants were APOε4+. When analyzing the subgroup that received MCT oil in the second four-month crossover period then continued MCT supplementation during the seven-month open-label extension, the authors reported that uninterrupted use of MCT oil for a total of 11 months (i.e., the placebo-MCT arm in the crossover trial) was associated with significant improvements in Cognigram^®^ 1 cognitive scores. A closer look at baseline values, however, reveals that the placebo-MCT arm actually had significantly higher Cognigram^®^ 1 scores at baseline, possibly influencing results. When baseline values are included in the comparison, the placebo-MCT arm experienced a 1.47% increase in Cognigram^®^ 1 scores from baseline to study conclusion, while the MCT-placebo arm experienced a 19.97% increase in Cognigram^®^ 1 scores during the same timeframe. While these results are encouraging for future research, they would be categorized as Class III evidence given the presence of non-equivalent baseline characteristics.

Other studies provided further evidence supporting the efficacy of ketogenic interventions in AD, though potential modulating effects of APOε genotype were frequently not assessed (25, 26, 28, 29). Collectively, ketogenic interventions in AD are supported by one Class I study and three Class II studies. This justifies a “B” (probably effective) recommendation under AAN criteria, with the caveat that this would only apply to patients who are APOε4-. Among patients who are APOε4+, the current data justifies a “U” (data inadequate or conflicting) recommendation under AAN criteria. This may change in the near future as one additional Class III study would justify a “C” (possibly effective) recommendation, at least regarding evidence of cognitive stabilization (rather than improvement *per se*).

### 4.2. Mild cognitive impairment

The multi-phase BENEFIC trial provided some of the strongest evidence to date supporting the use of ketogenic interventions in patients with MCI (35, 36). In this trial, 122 subjects with MCI were randomized to receive either a ketogenic MCT formula or placebo daily for 6 months. Supplementation with the ketogenic MCT formula was associated with significant improvements in cognitive tests compared to placebo. No significant effect of APOε4 status on ketosis or cognitive outcomes was observed, though the study was not adequately powered to assess APOε subgroups. This trial is categorized as Class II evidence, as the completion rate for the protocol was 68%.

The BENEFIC trial was preceded by a small study of 23 older adults with MCI conducted by Krikorian et al. (34). Subjects were randomized to either a ketogenic diet or a high-carbohydrate diet for 6 weeks, with the ketogenic diet being associated with significantly improved verbal memory (34). Another small trial conducted by Neth et al. (19) involved a randomized crossover design comparing a modified Mediterranean-Ketogenic diet to a low-fat American Heart Association (control) diet among 20 subjects with subjective memory complaints or MCI (19). Memory improved with both diets, though this may be attributable to practice effects. Compared to the control diet, a modified Mediterranean-Ketogenic diet was associated with significant improvements in the Free and Cued Selective Reminding Test, whereas similar effects were not observed for total story recall and ADAS-Cog12 outcomes. The modified Mediterranean-Ketogenic diet was associated with significant improvements in peripheral metabolic profile, cerebral perfusion, and cerebral ketone body uptake as compared to the control diet. Collectively, more data is needed to assess ketogenic interventions in MCI (particularly to assess the potential modulating role of APOε4 status) but the existing literature justifies a “B” (probably effective) recommendation per AAN criteria in light of multiple consistent Class II studies.

### 4.3. PD

A small (*n* = 7) proof-of-concept study assessing the efficacy of a ketogenic diet for patients with PD was conducted by VanItallie et al. (2). Five out of seven participants completed the protocol, with all five completers showing improved total UPDRS scores and improved motor subscores. This study achieved remarkably high levels of ketonemia among the three most adherent subjects (mean serum β-HB concentration of 6.6 mmol/L). In 2018, Phillips et al. (41) conducted a larger trial using a less stringent ketogenic diet as compared to the diet employed by Vanittalie et al. (2). 47 subjects with PD were randomized to consume either the modified ketogenic diet or a low-fat (control diet) for 8 weeks. Mean serum β-HB concentration increased to 1.15 mmol/L in the ketogenic group. The ketogenic group demonstrated a 41% improvement in UPDRS I scores (non-motor daily living experiences), as compared to an 11% improvement in the control group, with the largest between-group differences observed for fatigue, daytime sleepiness, pain and other sensations, urinary problems, and cognitive impairment. A randomized controlled pilot trial conducted by Krikorian et al. (42) compared a ketogenic diet to a control diet among 14 individuals with MCI in the setting of PD (42). Mean serum β-HB concentration increased to 0.31 mmol/L in the ketogenic group. Relative to the control group, the ketogenic group demonstrated significant improvements in memory. No significant improvements in motor symptoms were observed.

More recently, Norwitz et al. (40) performed a randomized, placebo-controlled, crossover study in 14 subjects with PD, comparing the effects of acute administration of a ketone ester drink and a taste-matched, carbohydrate-based control drink on endurance exercise performance (40). The ketone ester drink raised participants' β-HB level to a mean of 3.5 mmol/L within 30 min of consumption. Consumption of the ketone ester drink was associated with a 24% increase in exercise endurance capacity as compared to the isocaloric placebo drink, suggesting that ketone ester supplementation has the potential to modulate motor performance in PD.

More research is needed to elucidate the potential for ketogenic interventions to alleviate motor features of PD, particularly given the varying degrees of ketonemia achieved with differing protocols (as detailed above). Based on limited preliminary data, it is possible that more potent formulations (and correspondingly higher circulating β-HB levels) may be needed to significantly influence motor features. It is also possible that multi-pronged interventional approaches (of which therapeutic ketosis may represent a single dimension) may be required to address the complexity of PD pathophysiology (50). This line of research currently supports a “C” (possibly effective) recommendation for non-motor symptoms and a “U” recommendation (data inadequate or conflicting) for motor symptoms in PD based on AAN criteria. With promising preliminary findings and multiple clinical trials in the pipeline, however, this may soon evolve in the coming years.

### 4.4. Effects of therapeutic ketosis on biomarker outcomes

Although most of the reviewed studies demonstrating cognitive and/or motor symptomatic improvement may (at least in part) support the “alternative fuel” hypothesis, there is limited evidence suggesting that therapeutic ketosis may favorably affect AD-specific biomarkers in patients at risk for AD. Although not all biomarker outcomes featured in the reviewed studies reached significance (38), Neth et al. (19) found that therapeutic ketosis favorably affected CSF Aβ42, CSF tau, and CSF neurogranin in patients at risk for AD (19). These presumed disease-modifying (i.e., preventive) effects suggest (at least in part) support for the signaling hypothesis of therapeutic ketosis in neurodegeneration. However, more research is needed to validate this hypothesis and identify optimal biomarkers for clinical translation.

In contrast to the more immediate symptomatic effects associated with alternative fuel substrate provision in patients with advanced brain glucose metabolic deficits, the effects of such “signaling” mechanisms may follow a delayed time course over weeks to months or longer, as such effects will be mediated through signaling pathways and epigenetic changes as opposed to more immediate substrate-level ‘fuel' availability (14, 51, 52). Although the reviewed studies demonstrated the possibility of cognitive improvement in MCI (36), it is also possible that mitigation, slowing, or even prevention of disease progression may be seen rather than more immediate cognitive improvement. This may necessitate future study designs with primary outcome measures (e.g., clinical severity ratings vs. use of proxy biomarkers) that differ between late vs. early stages of neurodegenerative disease. If this line of reasoning regarding therapeutic ketosis as a means of preventive medicine proves to be true, modification of disease trajectory may be proportionally greater when intervention is initiated in prodromal stages of neurodegeneration. Given the pleiotropic effects of therapeutic ketosis, various mechanisms will undoubtedly be at play in both early and late stages of neurodegeneration, though the relative rates and clinical importance of differing mechanisms may vary.

### 4.5. Further considerations

Several important themes emerged in our analysis of the existing literature. First, it is important to note the limitations of the AAN Classification of Evidence approach. While it provides an objective framework for assessing the strength of evidence for an intervention, it should be noted that this framework is optimally applied in the assessment of pharmaceutical interventions. In particular, a primary criterion for Class I evidence under AAN criteria is concealed allocation, which is often not achievable for a dietary intervention. As such, several high-quality studies that we reviewed were precluded from being classified as Class I evidence, with relatively stronger classifications applied to dietary supplements that can be compared to taste-matched placebo. In addition, the majority of clinical trials we identified were small studies limited in statistical power and treatment duration. Larger-scale, pivotal trials are justified in these populations to validate preliminary findings.

Another important point of consideration is the variety of ketogenic interventions utilized in differing study protocols. In the interest of providing objective criteria for comparison, we included the mean serum β-HB concentration measured among participants in Table 1 whenever possible, often noticing striking differences in the relative degrees of ketonemia achieved by various protocols. The possibility of a dose-response relationship for therapeutic ketosis depending on the clinical context offers an interesting hypothesis to guide future research.

In particular, the mixed preliminary results observed among subjects who are APOε4+ suggest the possibility of a dose-response curve applying to these patients. The strongest evidence supporting non-response to a ketogenic intervention among patients who are APOε4+ was provided by the Henderson et al. (24) trial, which reported a mean post-dose β-HB concentration in the range of 0.36–0.39 mmol/L for the experimental arm (24). For reference, we have included a table outlining clinical interpretations for respective degrees of ketonemia (see Table 3).

**Table 3:** Serum β-HB concentration: Respective clinical interpretation.

0.0–0.5 mmol/L	Generally considered below the threshold of significant ketosis, though some studies have demonstrated positive results with lower degrees of ketonemia (in particular among APOε4- individuals)
0.5–2.5 mmol/L	Generally considered an optimal range for the ketogenic diet
2.5–4 mmol/L	Generally considered an optimal range for a diet supplemented with exogenous ketones
>4 mmol/L	Generally considered supratherapeutic, with concern for side effects at higher doses

**Figure 1:**
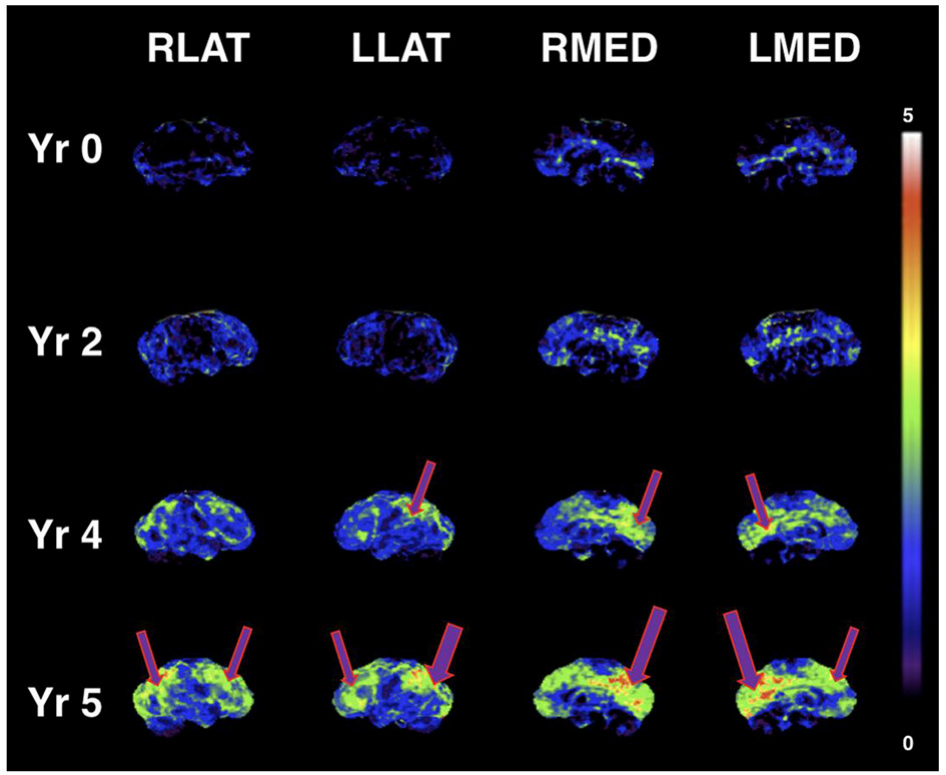
Serial FDG PET images showing progressive metabolic reductions from reference ranges at baseline (Yr 0), through stages of mild cognitive impairment (Yr 2 and Yr 4), and clinical diagnosis of dementia (Yr 5) in PD dementia converter. Cortical Z-score maps based on normal control data are shown (max Z-score is 5; higher scores represent more severe glucose metabolic deficits). Purple arrows represent reduced glucose metabolism. Similar to worsening metabolic deficits seen in MCI and AD, cortical glucose metabolic reductions become more severe and extensive with progression of cognitive decline (in this case, glucose uptake in key brain regions such as the posterior cingulum and precuneus is ~3–5 standard deviations lower than control data at Yr 5). According to the “alternative fuel” hypothesis, escalating dose or potency of ketogenic interventions may be needed for greater brain metabolic deficits (greater brain energy “gap”). Figure reproduced with permission from Bohnen et al. (54).

**Figure 2:**
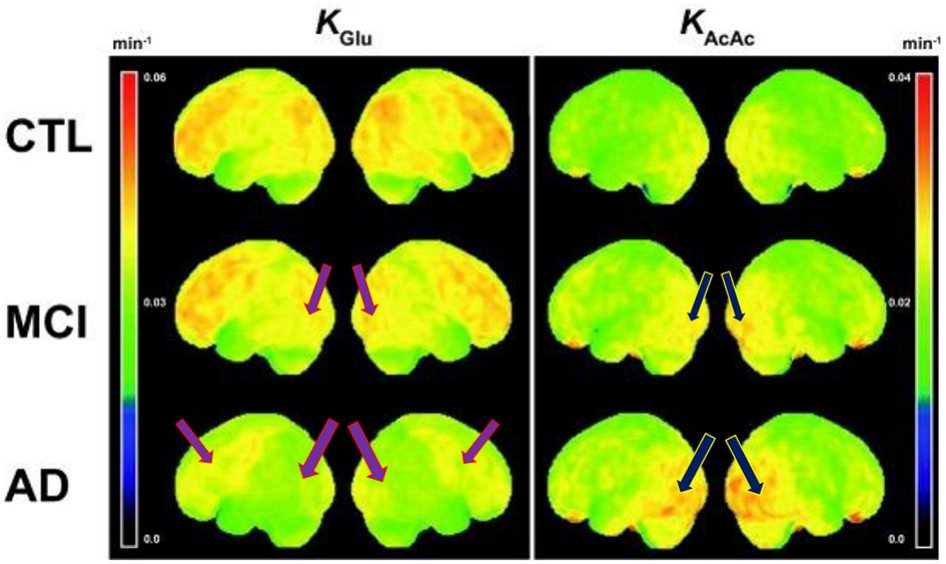
Voxel-wise 3D surface projection of the brain uptake rate constant for glucose (KGlu; min– 1) and acetoacetate (KAcAc; min– 1) in healthy older controls (CTL; *n* = 24), mild cognitive impairment (MCI; *n* = 20) and early Alzheimer's disease (AD; *n* = 19). Note the worsening reductions in glucose metabolism from control to MCI and most severe reductions in AD (left column images). Despite these glucose metabolic reductions, there is preserved to even increased ketone body uptake (right column images), that is more prominent in AD compared to MCI. Purple arrows represent reduced glucose metabolism and blue arrows represent increased ketone metabolism. These findings indicate that reduced glucose metabolism in MCI and AD still reflects the presence of viable neurons and glial cells. Figure reproduced with permission from Croteau et al. (9).

A case report published by Newport et al. (53) found that a patient with severe manifestations of AD who was APOε4+ responded clinically to a ketone ester intervention (53). The patient had initially improved during treatment with coconut oil and medium-chain triglyceride (caprylic and capric) fatty acids, as his MMSE score had improved from 12 to 20; his Activities of Daily Living (ADL) score had risen 14 points; and he had exhibited gradual improvement in gait, social participation, and memory. However, his clinical condition deteriorated while participating in a clinical trial assessing the γ-secretase inhibitor, semagacestat. Increasingly desperate for a remedy, his wife (a physician who had assumed his caregiving responsibilities) suggested a trial of a ketone ester intervention (a more potent formulation as compared to coconut oil and medium-chain triglycerides). Subsequently, the patient began augmenting the coconut oil and medium-chain triglyceride regimen with high doses of exogenous ketones (starting at 21.5 g of [R]-3-hydroxybutyl [R]-3-hydroxybutyrate monoester three times daily [TID], then titrating to 28.7 g TID), which was associated with marked clinical improvement. Of note, post-dose serum β-HB measurements obtained during the ketone ester intervention period ranged from 3 mmol/L to 7 mmol/L. This case study suggests evidence of a possible dose-response function that may be dependent on the severity of cognitive impairment, the latter being also a function of more severe glucose metabolic deficits in the brain. Figure 1 illustrates the progressive glucose metabolic deficits that occur during the disease trajectory from normal cognition (few cortical deficits) to mild cognitive impairment (mild-to-moderate deficits) to dementia (severe and extensive deficits) with serial FDG PET scans obtained over 5 years in a different case study of a patient with PD. Similarly, Figure 2 demonstrates more severe glucose metabolic deficits associated with progression from normal cognition to AD, while ketone metabolism pathways are preserved or even up-regulated in the presence of glycolytic dysfunction.

It is thus possible that patients who are APOε4+ may require relatively higher degrees of ketosis (i.e., more potent interventions) to achieve clinically significant outcomes. Table 4 provides a conceptual outline for the stepwise progression of ketogenic intervention potency, with the crucial caveat that more may not always be better. Greater degrees of ketonemia necessitate greater safety precautions, as higher levels may bear greater potential for adverse effects. Attention should also be paid to the half-life of more potent interventions, as pulsatile vs. more sustained levels of ketosis may have differing effects on human physiology.

**Table 4:** Relative potency of ketogenic interventions.

**Intervention**	Typical serum β-HB level^*^
12-hour fast	0.08–0.1 mmol/L
Coconut oil	0.2–0.3 mmol/L
MCT oil	0.3–0.6 mmol/L
Low-carbohydrate diet	0.4–0.65 mmol/L
Ketogenic diet	0.5–2.5 mmol/L
Exogenous ketones	2.5–4 mmol/L

* While serum β-HB concentration will vary as a function of dosage as well as potency in the setting of supplementation, these ranges were approximated in the process of our literature review as being generally representative of the mean serum β-HB level achieved by subjects in response to a particular intervention. We also referred to data published by Henderson et al. (24) and Norgen et al. (55).

Conceptualizing therapeutic potency solely as a function of serum β-HB level, however, fails to capture the complexity of intersecting factors influencing metabolic outcomes. Perhaps most pertinently, it is widely known that ketone metabolism is altered in the setting of high glycemic intake with resultant elevations in insulin signaling. Post-mortem brain studies of young adults who are APOε4+ have shown evidence of not only glycolytic enzyme and mitochondrial dysfunction, but also ketone metabolism pathway changes with evidence of mixed compensatory and failing elements (56). Therefore, it is possible that a combined low-glycemic intake and ketone body supplementation approach may be required in these patients as, otherwise, continuation of high glycemic intake may further compromise mitochondrial function, thereby limiting mitochondrial capacity for optimal ketone body oxidation. There is evidence that APOε4+ status may be a risk factor for not only dysglycemia but also for dysfunctional PPAR-γ/PGC-1α signaling pathway functions, resulting in altered mitochondrial biogenesis (23). This observation bears clinical relevance as the potential effectiveness of a ketogenic intervention in APOε4+ carriers to bypass a block in the glycolytic pathway may be reduced if a block in mitochondrial oxidation capacity is also present.

Lastly, an important point of consideration involves long-term risks and safety, as most of the studies identified were of short duration (with intervention periods generally ranging from 3 weeks to 3 months). Notable exceptions include the 15-month Juby et al. (33) crossover study and the six-month BENEFIC trial, both of which assessed MCT supplementation. Further studies are thus needed to assess the long-term safety of ketogenic interventions, particularly for more potent exogenous ketone formulations and strict ketogenic diet protocols.

## 5. Conclusions and future directions

Ketogenic interventions are probably effective for cognitive improvement in patients with mild-to-moderate AD who are APOε4- and in patients with MCI. Albeit with methodological limitations, current evidence to date suggests that patients who are APOε4+ may derive relatively less benefit (if any at all) from ketogenic interventions, at least when dosed equivalently to patients who are APOε4- and when using lower-potency ketogenic interventions, such as medium-chain triglycerides. Based on limited preliminary data, it is plausible that modified ketogenic protocols (e.g. more potent interventions) may be required for patients with AD who are APOε4+ to achieve clinically significant benefits. Similarly, more potent ketogenic interventions may be needed with advancing level of cognitive impairment in both AD and PD given the postulated “brain energy gap” (i.e., discrepancy of abnormal glucose metabolism versus preserved-to-upregulated ketone body metabolism observed in brain PET studies). Furthermore, assessments of possible downstream mitochondrial oxidation defects may be important to identify ketogenic intervention responders from non- or poor responders. Lastly, effects of ketogenic interventions may not only depend on primary brain (or body) cellular energetic effects (‘bioenergetic' hypothesis) but also on brain and body signaling functions (‘signaling' hypothesis) and even microbiome factors, which may require a specific set of additional biomarkers to best gauge outcome effects. Assessment of glycemic functions is also recommended, as it remains unclear to what extent clinical benefits associated with ketogenic diets can be directly attributable to ketosis versus a reduction in glucose and insulin metabolism. This may be of particular relevance for patients with AD who are APOε4+ given the possibility that a combined low-glycemic intake and ketone body supplementation approach may be required in this clinical setting (i.e. continuing high glycemic intake may further compromise mitochondrial function, thereby limiting mitochondrial capacity for ketone body oxidation). In summary, we recommend the following directions for future research:

1. Future studies should consistently evaluate APOε4 status and stratify accordingly.
2. Differing ketogenic protocols may be needed as predicted by emerging biomarkers and differing clinical contexts, with future research further delineating optimal utilization of therapeutic ketosis *via* a precision medicine approach. Standardization of protocols may be guided by target levels/durations of ketonemia among other metrics. Future study designs assessing disease-modifying or preventive effects of therapeutic ketosis may require primary outcome measures (e.g. clinical severity ratings vs. use of proxy biomarkers) that differ between late vs. early stages of neurodegenerative disease.
3. In particular, glycemic and mitochondrial biomarkers may prove to be essential in predicting clinical response to ketogenic interventions.
4. Adequately powered, pivotal trials are justified in these populations to validate preliminary findings, with a focus on clinically meaningful endpoints (or at least those recognized as relevant by regulatory authorities).
5. Neuroimaging correlates (e.g. fMRI and PET) should be utilized to provide further mechanistic evidence.
6. Future studies should also assess relative effects of pulsatile vs. more sustained levels of ketosis, particularly if ketosis is sustained for long durations and mixed with glycemic intake (e.g. long-term ketone ester supplementation in the setting of a regular diet).
7. Importantly, long-term studies are needed to assess safety, compliance, and long-term effects of ketogenic interventions.

**Figure 3:**
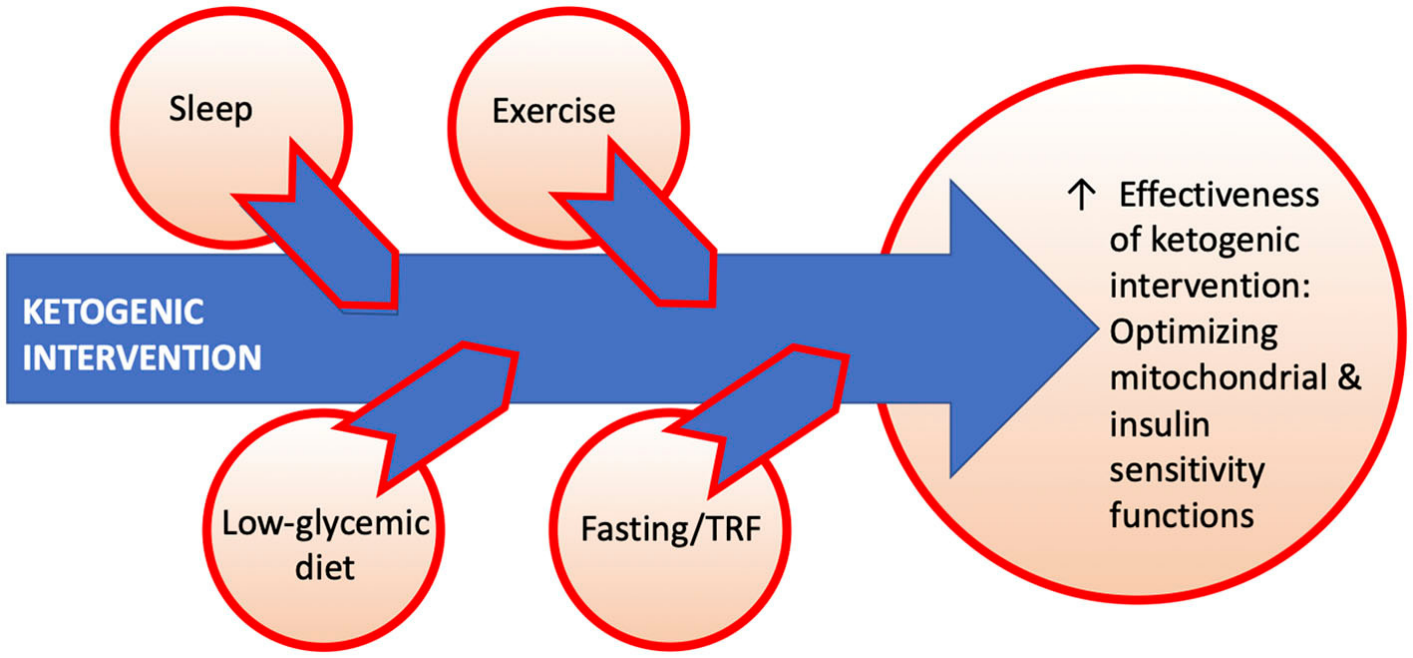
Hypothesized conceptual model of actionable sleep, exercise, diet, and fasting/time-restricted feeding (TRF) lifestyle factors that may augment effects of ketogenic interventions.

Taking all considerations into account, we recognize the potential for a multidimensional model geared toward optimizing the effectiveness of ketogenic interventions as dictated by clinical setting. Figure 3 shows this hypothesized conceptual model of actionable sleep, exercise, diet, and fasting/time-restricted feeding (TRF) lifestyle factors that may augment effects of ketogenic interventions. Common mechanistic elements include mitochondrial biogenesis (esp. sleep, exercise) and insulin sensitivity (exercise, low-glycemic intake, fasting, TRF) (57). Slow-wave sleep in particular may play a modulatory role in CNS glycolytic functions and clearance of cerebral metabolic waste products (58, 59). These (and other) potentially complementary interventions may have different applications in different stages of neurodegenerative disorders or disease-specific applications in targeted diseases. Importantly, future research is needed to investigate this proposed model.

## Data availability statement

The original contributions presented in the study are included in the article/Supplementary material. Further inquiries can be directed to the corresponding author.

## Author contributions

JB drafted the main text and tables based on recommendations derived from conceptual discussions with the co-authors, after which RA wrote the bulk of the introduction discussing neuroimaging evidence to date and provided further edits and suggestions throughout the entirety of the document. NB drafted the abstract, the first section of the introduction discussing the clinical history of therapeutic ketosis, and the conclusion, in addition to providing edits and suggestions throughout the entirety of the document as well as playing a key role in identifying relevant clinical studies to be included in the systematic review. All authors contributed to the article and approved the submitted version.

## Supplementary material

Supplementary Data Sheet 1 Flow diagram.

Supplementary Data Sheet 2 American Academy of Neurology classification of evidence.
