# Supplementary material for: Ketogenic interventions in mild cognitive impairment, Alzheimer's disease, and Parkinson's disease: A systematic review and critical appraisal
Source: Front Neurol. 2023 Feb 9;14:1123290. doi: 10.3389/fneur.2023.1123290 (PMC9947355; doi:10.3389/fneur.2023.1123290)
Supplement: Supplementary Data Sheet 1 — Flow diagram. [file Data_Sheet_1.pdf]

Ketogenic Interventions in MCI, AD, and PD: A systematic review and critical appraisal (flow diagram)

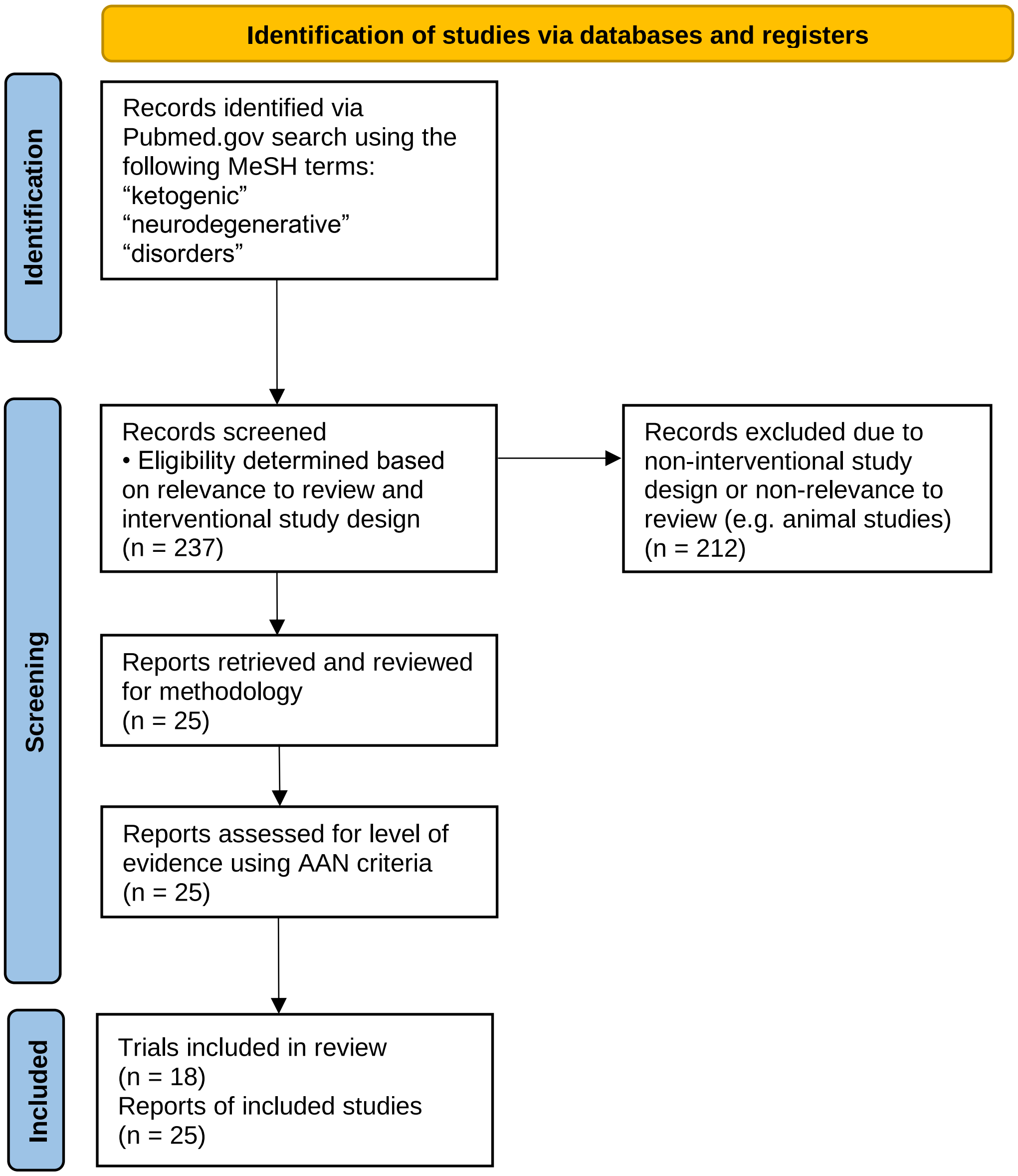

<u>Abbreviations</u>:
AAN: American Academy of Neurology
AD: Alzheimer disease
MCI: Mild Cognitive Impairment
MeSH: Medical Subject Headings
PD: Parkinson disease
