# Supplementary material for: Ketogenic interventions in mild cognitive impairment, Alzheimer's disease, and Parkinson's disease: A systematic review and critical appraisal
Source: Front Neurol. 2023 Feb 9;14:1123290. doi: 10.3389/fneur.2023.1123290 (PMC9947355; doi:10.3389/fneur.2023.1123290)
Supplement: Supplementary Data Sheet 2 — American Academy of Neurology classification of evidence. [file Data_Sheet_2.pdf]

American Academy of Neurology Classification of Evidence

Therapeutic

Class I: A randomized, controlled clinical trial of the intervention of interest with masked or objective outcome assessment, in a representative population. Relevant baseline characteristics are presented and substantially equivalent among treatment groups or there is appropriate statistical adjustment for differences.

The following are also required:

a. concealed allocation
b. primary outcome(s) clearly defined
c. exclusion/inclusion criteria clearly defined
d. adequate accounting for drop-outs (with at least 80% of enrolled subjects completing the study) and cross-overs with numbers sufficiently low to have minimal potential for bias.
e. For non-inferiority or equivalence trials claiming to prove efficacy for one or both drugs, the following are also required*

1. The authors explicitly state the clinically meaningful difference to be excluded by defining the threshold for equivalence or non-inferiority.
2. The standard treatment used in the study is substantially similar to that used in previous studies establishing efficacy of the standard treatment. (e.g. for a drug, the mode of administration, dose and dosage adjustments are similar to those previously shown to be effective).
3. The inclusion and exclusion criteria for patient selection and the outcomes of patients on the standard treatment are comparable to those of previous studies establishing efficacy of the standard treatment.
4. The interpretation of the results of the study is based upon a per protocol analysis that takes into account dropouts or crossovers.

Class II: A randomized controlled clinical trial of the intervention of interest in a representative population with masked or objective outcome assessment that lacks one criteria a-e above or a prospective matched cohort study with masked or objective outcome assessment in a representative population that meets b-e above. Relevant baseline characteristics are presented and substantially equivalent among treatment groups or there is appropriate statistical adjustment for differences.

Class III: All other controlled trials (including well-defined natural history controls or patients serving as own controls) in a representative population, where outcome is independently assessed, or independently derived by objective outcome measurement.**

Class IV: Studies not meeting Class I, II or III criteria including consensus or expert opinion.

*Note that numbers 1-3 in Class Ie are required for Class II in equivalence trials. If any one of the three are missing, the class is automatically downgraded to Class III.

**Objective outcome measurement: an outcome measure that is unlikely to be affected by an observer's (patient, treating physician, investigator) expectation or bias (e.g., blood tests, administrative outcome data).

Diagnostic

Class I: A cohort study with prospective data collection of a broad spectrum of persons with the suspected condition, using an acceptable reference standard for case definition. The diagnostic test is objective or performed and interpreted without knowledge of the patient's clinical status. Study results allow calculation of measures of diagnostic accuracy.

Class II: A case control study of a broad spectrum of persons with the condition established by an acceptable reference standard compared to a broad spectrum of controls or a cohort study where a broad spectrum of persons with the suspected condition where the data was collected retrospectively. The diagnostic test is objective or performed and interpreted without knowledge of disease status. Study results allow calculation of measures of diagnostic accuracy.

Class III: A case control study or cohort study where either persons with the condition or controls are of a narrow spectrum. The condition is established by an acceptable reference standard. The reference standard and diagnostic test are objective or performed and interpreted by different observers. Study results allow calculation of measures of diagnostic accuracy.

Class IV: Studies not meeting Class I, II or III criteria including consensus, expert opinion or a case report.

Prognostic

Class I: A cohort study of a broad spectrum of persons at risk for developing the outcome (e.g. target disease, work status). The outcome is defined by an acceptable reference standard for case definition. The outcome is objective or measured by an observer who is masked to the presence of the risk factor. Study results allow calculation of measures of prognostic accuracy.

Class II: A case control study of a broad spectrum of persons with the condition compared to a broad spectrum of controls or a cohort study of a broad spectrum of persons at risk for the outcome (e.g. target disease, work status) where the data was collected retrospectively. The outcome is defined by an acceptable reference standard for case definition. The outcome is objective or measured by an observer who is masked to the presence of the risk factor. Study results allow calculation of measures of prognostic accuracy.

Class III: A case control study or a cohort study where either the persons with the condition or the controls are of a narrow spectrum where the data was collected retrospectively. The outcome is defined by an acceptable reference standard for case definition. The outcome is objective or measured by an observer who did not determine the presence of the risk factor. Study results allow calculation of measures of a prognostic accuracy.

Class IV: Studies not meeting Class I, II or III criteria including consensus, expert opinion or a case report.

Screening

Class I: A statistical, population-based sample of patients studied at a uniform point in time (usually early) during the course of the condition. All patients undergo the intervention of interest. The outcome, if not objective, is determined in an evaluation that is masked to the patients' clinical presentations.

Class II: A statistical, non-referral-clinic-based sample of patients studied at a uniform point in time (usually early) during the course of the condition. Most patients undergo the intervention of interest. The outcome, if not objective, is determined in an evaluation that is masked to the patients' clinical presentations.

Class III: A sample of patients studied during the course of the condition. Some patients undergo the intervention of interest. The outcome, if not objective, is determined in an evaluation by someone other than the treating physician.

Class IV: Studies not meeting Class I, II or III criteria including consensus, expert opinion or a case report.

Causation

Class I: Prospective cohort study design that satisfies these criteria— (a) groups studied are representative of population of interest ('broad spectrum'); (b) risk factors and outcomes are clearly defined with validated or generally accepted criteria, and measured independently or objectively; (c) comparison groups are matched for known possible confounding risk factors, or the effects of such confounders are controlled in the study analysis; AND (d) measures of association are expressed (or can be calculated) as rate ratios, risk ratios, relative risks (R.R.) or population attributable risks with confidence intervals.

Class II: Retrospective cohort or case-control study designs that satisfy criteria (a), (b), and (c) above, in which (d) the measure of association may also be expressed (or can be calculated) as an odds ratio (O.R.) with confidence intervals.

Class III: Other cohort or case-control study designs in which groups studied represent a narrow spectrum of the population of interest, or the measure of association does not include an R.R. or O.R. but does include an aggregate measure such as a correlation or group mean with standard deviation or p-value. Criterion (b) above must still be satisfied. Obvious confounding is not evident.

Class IV: Studies not meeting criteria for Class I, II, or III. Specifically, studies that are non-comparative, unrepresentative of the population of interest, with major biases or confounding, lacking useful measures of effect, or lacking measures of effect estimate stability.

Notes:

1. In addition to the criteria above, any causal inference requires that exposure to the risk factor precede the development of the outcome. In addition, there may be need to allow for an induction period.
2. In translating evidence, a requirement of two or more studies implies that such studies should not include the same subjects.
3. Exploratory studies involving multiple comparisons of a variety of exposures and outcomes may be rated lower if it is evident that the study was designed without an *a priori* hypothesis or focus upon the specific exposure and outcome of interest.
4. Randomized clinical trials (RCTs) are equivalent to prospective cohort studies in which the risk of confounding has been minimized. Evidence from such studies may be considered Class I, provided it satisfies criteria (a), (b), and (d) above. Note, however, that it is preferable to apply the AAN criteria for therapeutic studies when classifying evidence pertaining to the experimental (treatment) variables of an RCT.

Classification of Recommendations

A = Established as effective, ineffective or harmful (or established as useful/predictive or not useful/predictive) for the given condition in the specified population. (Level A rating requires at least two consistent Class I studies.)*

B = Probably effective, ineffective or harmful (or probably useful/predictive or not useful/predictive) for the given condition in the specified population. (Level B rating requires at least one Class I study or two consistent Class II studies.)

C = Possibly effective, ineffective or harmful (or possibly useful/predictive or not useful/predictive) for the given condition in the specified population. (Level C rating requires at least one Class II study or two consistent Class III studies.)

U = Data inadequate or conflicting; given current knowledge, treatment (test, predictor) is unproven.

*In exceptional cases, one convincing Class I study may suffice for an "A" recommendation if 1) all criteria are met, 2) the magnitude of effect is large (relative rate improved outcome > 5 and the lower limit of the confidence interval is > 2).

Classification of Recommendations (causation)

A = Risk factor is a highly probable contributor to the development of disease or outcome. (Level A rating requires two or more consistent Class I studies all showing an effect size (R.R.) ≥ 2 with lower confidence limits >1. In addition, either (1) a causal inference is coherent with known biologic mechanisms and related scientific evidence or (2) findings clearly demonstrate that higher doses of exposure increase likelihood of disease or outcome.)

B = Risk factor is a probable contributor to the development of disease or outcome. (Level B rating requires at least one Class I study fulfilling other criteria above, OR two or more consistent Class II studies, showing an effect size (R.R. or O.R.) ≥ 1.5 with lower confidence limits >1.)

C = Risk factor is a possible contributor to the development of disease or outcome. (Level C rating requires 1 Class II or 2 or more Class III studies, showing effect estimate(s) with consistent significant departure(s) from null value.)

U = A causal relationship between the risk factor and disease or outcome is unproven or unsupported.
